# State Medical Convention

**Published:** 1850-03

**Authors:** 


### ARTICLE III.

### STATE MEDICAL CONVENTION.

We hope our readers in Illinois are alive to the importance of securing a large attendance at the first State Medical Convention ever held in the "Prairie State." It can scarcely be necessary to urge upon every one of our readers the propriety of making calculations to be present if possible. A failure to have an assemblage respectable in point of numbers, next June, may result in discouraging those who would take an active part in the matter, and thus retard an association so much needed, and one which, if properly conducted, would confer so many benefits on the profession.

It any thing effectual is to be done towards elevating the standard of professional attainment, the effort must proceed from a source entitled to the obedience and respect of every reputable physician in the State. If restraints are to be thrown around the ignorant and soulless charlatans who pilfer from the pockets, and trifle with the lives, of the unsophisticated, the movement must be made by the entire profession. If the bickerings and jealousies, which bring discredit and disgrace, upon us, by lowering us in the estimation of respectable communities, are to be adjusted by reference to a code of ethics, that code must be one formed by representatives of the whole Medical Fraternity. In brief, if any of the objects interesting and important to a liberal and learned profession are to be attained, an effort in that behalf must be made by every right minded, intelligent physician. Reforms can only be accomplished by a concert of action; and hereafter we hope to hear no more complaint, from those who are too indolent or indifferent to lend their strength to the aid of those already at work in the good cause, that the onward progress of our profession is checked. We conclude then: let every physician in Illinois endeavor to make arrangements to meet his brethren at Springfield on the first Tuesday in June, to take council on matters of the highest importance to every member of the profession; and at least, let every organized society within the State send delegates who will be the exponents of the wishes and feelings of those whom they represent.

Of course these remarks will apply with as much force to any other State, as to Illinois. In Indiana, a State organization was effected last year, and we doubt not the ensuing meeting will be more largely attended, and productive of much good to the profession in that State. The next meeting will be held on the Third Wednesday in May.

In Wisconsin a State Society has been organized. Its time of meeting we do not know. We will endeavor to learn before our next number shall be issued. In Michigan we believe there are two State Associations. In Iowa, so far as we know, no organization has been effected. We hope our friends over there will move in the matter. M.

